# Prosthetically guided oral implant surgery. A retrospective cohort study evaluating the 5-year surgical outcome

**DOI:** 10.3205/iprs000176

**Published:** 2023-08-18

**Authors:** Andreas Sakkas, Stefan Westendorf, Oliver Christian Thiele, Alexander Schramm, Frank Wilde, Sebastian Pietzka

**Affiliations:** 1 Department of Cranio-Maxillo-Facial-Surgery, German Armed Forces Hospital Ulm, Germany; 2 Department of Cranio-Maxillo-Facial-Surgery, University Hospital Ulm, Germany; 3 Department of Oral and Plastic Maxillofacial Surgery, Ludwigshafen Hospital, Ludwigshafen, Germany

**Keywords:** computer-assisted planning, guided implant surgery, autologous bone augmentation, dental implants

## Abstract

**Purpose:**

This study primarily evaluated the 5-year implant survival and success rate of prosthetically guided inserted implants. The secondary aim was to evaluate the impact of clinical variables on the development of mucositis, peri-implant bone resorption, peri-implantitis, as well as early and late implant failure.

**Materials and methods:**

An observational retrospective single-centre study was conducted on patients who were treated with dental implants in the department of oral and plastic maxillofacial surgery of the military hospital of Ulm University between 2008 and 2010. In all patients, computer-assisted 3D planning after wax-up of the prosthetic restoration and template-guided surgery with titanium implants were performed. Bone augmentation procedures were performed primarily if needed. Intraoperative and postoperative complications as well as technical and mechanical complications after prosthesis loading were evaluated. In a 5-year clinical and radiological follow-up, implant success and implant survival were assessed using descriptive statistics. A multivariable regression analysis evaluated the potential impact of augmentation procedures, wound healing complications, smoking, history of periodontitis, and preoperative API (approximal plaque index) and SBI (sulcus bleeding index) values on peri-implant mucositis, peri-implant bone resorption, peri-implantitis, as well as early and late implant failure.

**Results:**

In this study, 466 implants in 283 patients were considered for inclusion, and sufficient data were obtained for analysis from 368 (78.9%) implants in 229 (80.9%) patients. An overall implant survival rate of 98.1% (n=361/368) at the 5-year follow-up was revealed. According to the success criteria of the study, the 5-year success rate was 97.04% (n=263/271). An early implant failure of 1.07% (n=5/466) was recorded. 48.2% of the implants were affected by peri-implant mucositis (n=122/253), while peri-implant bone resorption was detected in 21.7% of the radiologically examined implants (n=59/271). Fifteen cases of peri-implantitis (5.5%) were detected. Peri-implant bone resorption increased significantly after bone augmentation procedures (p=0.028). Wound healing complications after implantation significantly increased the prevalence of late implant failure in the maxilla (p<0.001). Peri-implant bone resorption and peri-implantitis were significantly more prevalent in smokers (p=0.022/p=0.043). Implants in patients with API>20% presented significantly higher rates of peri-implant mucositis (p=0.042). Wound healing complications after augmentation, history of periodontitis, and SBI>20% had no significant impact on the study parameters.

**Conclusions:**

The study confirms the reliability of prosthetically guided implant surgery, showing a high implant survival and success rate in a 5-year follow-up. Intraoperative complications and technical or mechanical complications after prosthesis loading remain within acceptable clinical limits. The rate of peri-implant mucositis, peri-implant bone resorption, and peri-implantitis was within the current literature range. Optimizing periodontal health and reducing smoking would improve the outcome. Further studies need to clarify the clinical indications and investigate the long-term surgical outcome of this treatment concept.

## Zusammenfassung

**Zielsetzung:**

Diese Studie bewertete in erster Linie die 5-Jahres-Überlebens- und Erfolgsrate von prothetisch-geführt inserierten Implantaten. Das sekundäre Ziel bestand darin, den Einfluss klinischer Variablen auf die Entwicklung von Mukositis, periimplantärer Knochenresorption und Periimplantitis sowie frühes und spätes Implantatversagen zu evaluieren.

**Material und Methoden:**

: Eine retrospektive Beobachtungsstudie wurde an Patienten durchgeführt, die zwischen 2008 und 2010 in der Abteilung für Mund-, Kiefer- und Plastische Gesichtschirurgie des Bundeswehrkrankenhauses der Universität Ulm mit Zahnimplantaten behandelt wurden. Bei allen Patienten wurden nach dem prothetischen Wax-up eine computergestützte 3D-Planung und eine schablonengeführte Implantatinsertion durchgeführt. Bei Bedarf wurden primär Knochenaugmentationen durchgeführt. Intraoperative und postoperative Komplikationen sowie technische und mechanische Komplikationen nach prothetischer Restoration wurden evaluiert. In einem klinischen und radiologischen 5-Jahres-Follow-up wurden der Implantaterfolg und das Implantatüberleben anhand deskriptiver Statistik ausgewertet. Eine multivariable Regressionsanalyse bewertete den potenziellen Einfluss von Augmentationsverfahren, Wundheilungsstörungen, Rauchen, anamnestische Parodontitis und präoperativen API- (Approximal Plaque Index) und SBI-Werten (Sulcus Bleeding Index) auf periimplantäre Mukositis, periimplantäre Knochenresorption, Periimplantitis sowie frühen und späten Implantatverlust.

**Ergebnisse:**

In dieser Studie wurden 466 Implantate bei 283 Patienten berücksichtigt und von 368 (78,9%) Implantaten bei 229 (80,9%) Patienten wurden ausreichende Daten für die Analyse gewonnen. Bei der 5-Jahres-Nachbeobachtung ergab sich eine Gesamtüberlebensrate von 98,1% (n=361/368). Nach den Erfolgskriterien der Studie lag die 5-Jahres-Erfolgsrate bei 97,04% (n=263/271). Ein frühzeitiger Implantatvelust wurde bei 1,07% (n=5/466) verzeichnet. 48,2% der Implantate waren von periimplantärer Mukositis betroffen (n=122/253), während bei 21,7% der radiologisch untersuchten Implantate eine periimplantäre Knochenresorption festgestellt wurde (n=59/271). Fünfzehn Fälle von Periimplantitis (5,5%) wurden festgestellt. Die periimplantäre Knochenresorption nahm nach augmentativen Verfahren signifikant zu (p=0.028). Wundheilungsstörungen nach der Implantation erhöhten die Prävalenz des späten Implantatverlustes im Oberkiefer signifikant (p<0.001). Periimplantäre Knochenresorption und Periimplantitis waren bei Rauchern signifikant häufiger anzutreffen (p=0.022/p=0.043). Periimplantäre Mukositis trat signifikant häufiger bei Patienten mit API>20% auf (p=0.042). Wundheilungsstörungen nach Augmentation, anamnestische Parodontitis und SBI>20% hatten keinen signifikanten Einfluss auf die Studienparameter.

**Schlussfolgerungen:**

Die Studie bestätigt die Zuverlässigkeit der prothetisch-geführten Implantatchirurgie und zeigt eine hohe Implantatüberlebens- und Erfolgsrate in einem 5-Jahres-Follow-up. Intraoperative Komplikationen und technische oder mechanische Komplikationen nach prothetischer Belastung bleiben innerhalb akzeptabler klinischer Grenzen. Die Rate der periimplantären Mukositis, der periimplantären Knochenresorption und der Periimplantitis lag im Rahmen der aktuellen Literaturwerte. Die Optimierung der parodontalen Gesundheit und die Reduzierung des Rauchens würden das Outcome verbessern. Weitere Studien müssen die klinischen Indikationen klären und das chirurgische Langzeitergebnis dieses Behandlungskonzepts untersuchen.

## Background

The long-term success of oral implants in terms of functionality and aesthetic outcome depends on precise planning [1], [2], [3]. In recent years, 3D computer-assisted guided surgery has become increasingly important in dental implantology [1], [2], [3], [4], [5], [6], [7], [8], [9]. Computed tomography (CT) or cone-beam computed tomography (CBCT) scans are a prerequisite for accurate implant planning [10]. The benefits of guided implant insertion include increased predictability and decreased surgery time and complication rate [4]. 

The digital workflow of prosthetically driven planning for guided implant surgery has been developed to optimize clinical outcomes [11], [12], [13]. Not only the osseous anatomy but also the 3D planned implant-supported prosthesis with its final position and occlusal characteristics define the ideal implant placement [14]. Prosthetically guided implant treatment combines model analysis and 3D imaging and has been developed to create a patient-specific approach. This concept enables a correct implant position with favorable aesthetic outcome and thus facilitates optimal occlusion and implant loading achieving biomechanical and functional stability [14]. With dedicated software systems, the final prosthetic restoration is visualized virtually and the planned implant position is transferred into surgery with a virtually designed surgical template regarding the prosthetic set-up [4]. The prosthetically guided concept also has the advantage of a preoperative consideration of the residual alveolar bone anatomy and important anatomical structures, such as the inferior alveolar nerve and maxillary sinus. This facilitates the decision for pre-implantological alveolar ridge reconstruction or allows adequate implant placement when augmentative procedures are not possible, especially in edentulous or severely atrophied alveolar crests [1], [2], [4], [15], [16]. 

Different methods have been described to transfer the digital 3D plan to the intraoral situation and several navigation systems are available for guided implant surgery with controversial reports regarding their efficacy in the laboratory and clinical setting [2], [3], [17]. Surgical templates have been manufactured in different ways and mostly need advanced laboratory equipment. The extended number of software systems and their rapid evolution provides the clinician a wide variation to choose from. However, Colombo et al. in their systematic review reported that implant survival rate and effectiveness are similar for conventional and digital implant placement procedures, suggesting that research should focus more on identifying which clinical situations can benefit from implant-guided surgery [6]. Although the accuracy and reliability of prosthetically guided surgery have been well documented in the literature, few data exist concerning complication rate and medium-term implant success rate of this concept. 

The primary aim of this study was to investigate the medium-term surgical outcome of prosthetically guided inserted implants in terms of 5-year implant survival and success rate. The secondary aim was to evaluate the impact of clinical variables on the development of mucositis, peri-implant bone resorption, peri-implantitis, as well as early and late implant failure. 

## Methods

### Patient collection

For this retrospective cohort study, the records of all patients who underwent oral implantation in the department of oral and maxillofacial surgery at Ulm military hospital between October 2008 and March 2010 were reviewed. 

Records were retrieved from the hospital electronic database. Ethical approval for this study was obtained from the ethics committee of the chamber of physicians in Ulm University, Germany (Number: 85/14- Fa/se.). The study was performed in accordance with the Declaration of Helsinki 1964 and its later amendments (World Medical Association, Declaration of Helsinki). After individual consultation, patients gave written consent to clinical and, if necessary, radiological examination.

Patients who fulfilled the following inclusion criteria were enrolled:

1. Age ≥18 years
2. Implants planned for fully-guided insertion
3. Completed restorative and endodontic treatment prior to implant therapy
4. Healthy periodontal status, assessed at the initial clinical examination, with an approximal plaque index (API) ≤20% and sulcus bleeding index (SBI) ≤20% [18], [19]
5. Any necessary periodontal therapy completed
6. Any type of prosthetic rehabilitation planned

Exclusion criteria were:

1. No contact information given for recall examination
2. Inadequate medical charts

### Prosthetically guided implant planning

#### Preparation

**Figure 1:**
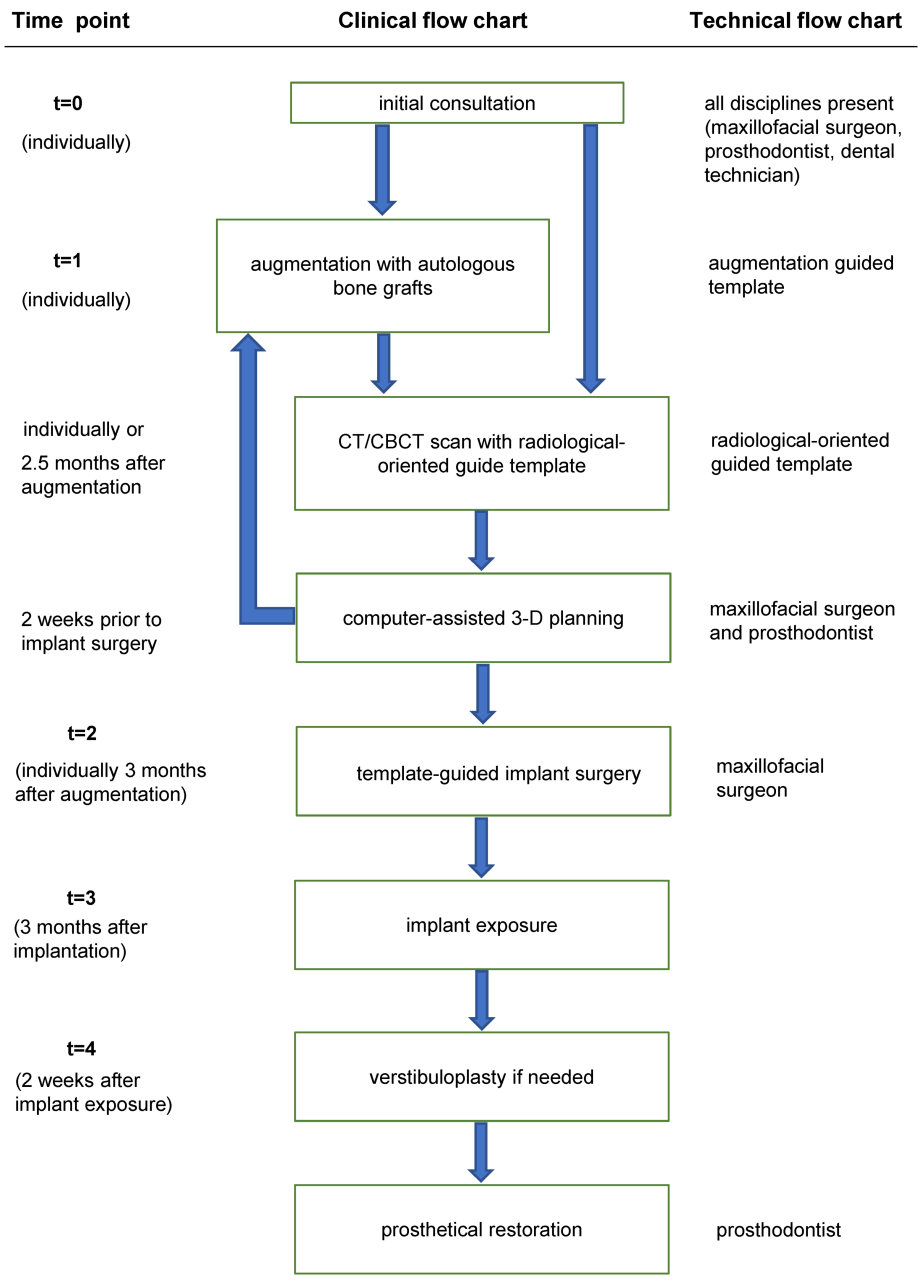
Flow chart of the prosthetically guided implant surgery concept t=time point, CT=computer tomography, CBCT=cone beam computer tomography

**Figure 2:**
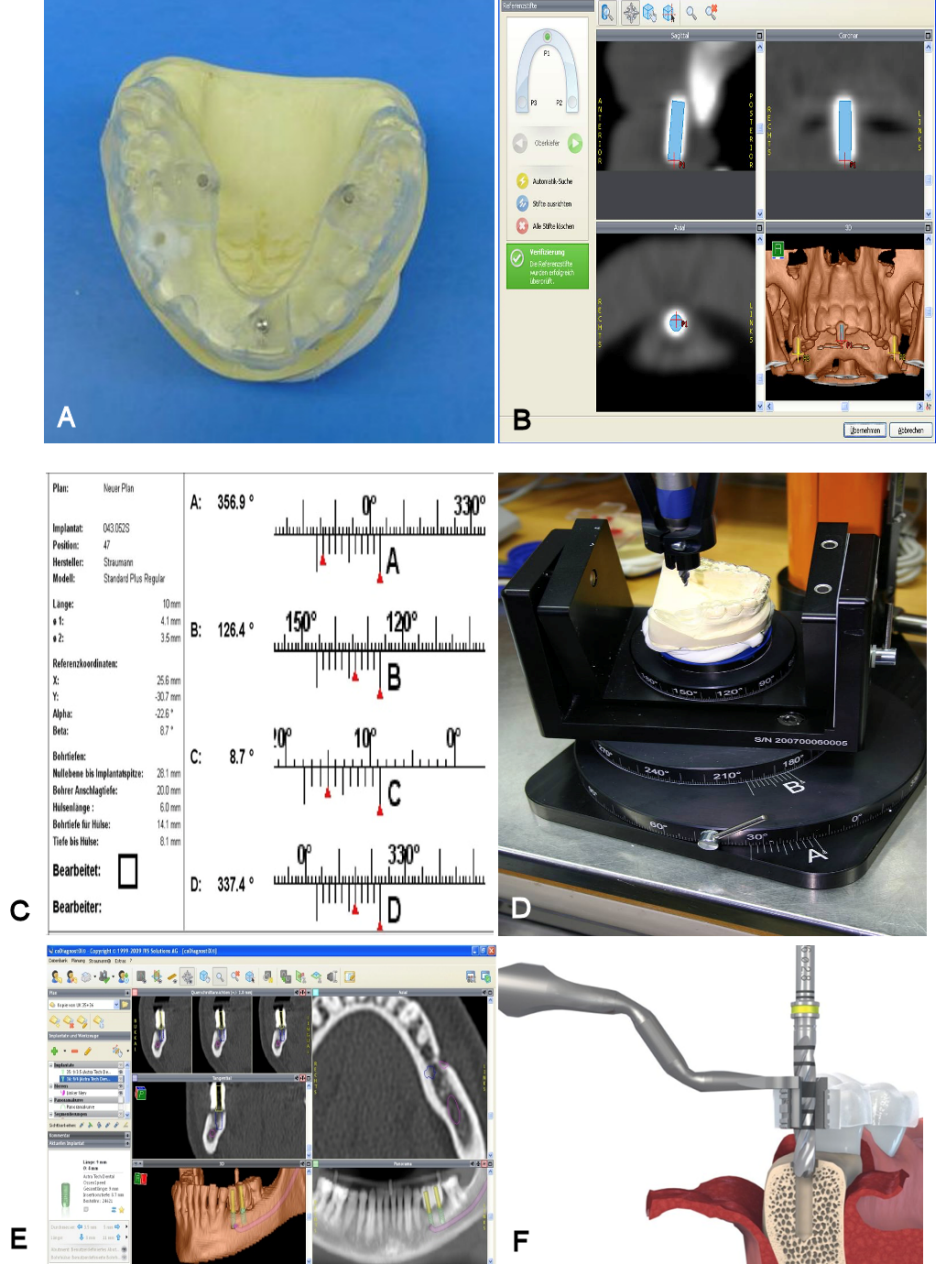
Technical way to manufacture the surgical drilling template, applying the prosthetically-guided planning concept by using digital planning software. A: Radiographic template with barium sulfate tooth 014 and the radiopaque markers. B: The radiopaque markers in the software display. The correction of the automatic detection is done manually if necessary. C: Planning data of an implant regio 047. On the left in the upper the selected implant system with the corresponding length and diameter can be seen. On the right and bottom left, the data important for fabricating the surgical guide are displayed. D: After setting the drilling table in 4 planes (A, B, C, D) according to the planning data, the drill hole is made in the drilling template. The metal die is then glued in place. E: Planning interface of the software. The implants were selected according to manufacturer, type, and diameter and placed in the jaw in the ideal prosthetic position shown by the X-ray template. The position data were sent to the laboratory for fabrication of the surgical guide. If there was no bone available, the bone augmentation was planned or, as in the present example, inserted paranervally to avoid costly vertical augmentations. F: Representation of the Straumann Guide System. Similar to the Steco system, however, here the drill sleeves are located on a guide tray to simplify handling. © Institut Straumann AG

The planning concept of the clinic is shown in Figure 1 (Fig. 1). The manufacture of the surgical drilling template using digital planning software is illustrated in Figure 2 (Fig. 2). The initial clinical examination was performed by an oral and maxillofacial surgeon, a prosthodontist from the clinic for dental prosthetics of the University of Ulm, and a master dental technician (Implantec^®^ laboratory, Amstetten, Germany). 

Occlusal and prosthetic analyses were performed intraorally and on plaster models at the initial examination. Standard diagnostic wax-ups were performed on articulated models in all cases. Following the wax-up of the planned prosthetic restoration, a scan template was created. Three titanium pins were inserted into the scan templates lingual to the tooth row for the spatial referencing of image data and image fusion. Interdental space, prospective implant axes, and prospective crown-implant length ratios were assessed based on the study models and panoramic radiographs. According to the tooth position in this wax-up, either a radiopaque template for the 3D radiograph or an augmentation template was fabricated [8]. 

The aim was to achieve “restitutio ad integrum” reconstruction in all patients. Indication for primary augmentation of the alveolar ridge defect was determined after computer-assisted 3D planning based on the following parameters:

- Presence of severe alveolar ridge atrophy rated class IV or V according to the Cawood and Howell classification [20]
- Residual maxillary bone <5 mm from the alveolar crest to the sinus floor

The donor site was chosen based on defect morphology and recipient site location. All bone harvesting procedures were performed using the same standardized surgical technique. Intraoral autologous bone block grafts were harvested using piezoelectric surgery from the lateral zygomaticoalveolar buttress, the ramus mandible in the retromolar area, and the symphysis mandible. Extraoral autologous bone block grafts from the inner surface of the iliac crest were harvested using oscillating saw and chisels.

#### Virtual planning

Computed tomography was performed in order to acquire three-dimensional image data sets for computer planning (Somatom Definition^®^ Siemens, Erlangen, Germany). The following scanner settings were used: tube potential 120 kV, tube current 230 mAs, increment 0.4 mm, rotation time 1s, collimation 0.6 mm. CT data sets were reconstructed at a slice thickness of 1 mm. If primary augmentation was primarily performed, the implant planning 3D scan was performed after a healing period of 3 to 4 months to assess bone volume.

Implant planning and simulation were performed with CoDiagnostiX^®^ software (version 6.0, IVS Solutions AG, Chemnitz, Germany). CT data were imported into the software in DICOM format, which was then used to virtually place implants into their position and to assess them in multiplanar (axial, coronal, and sagittal) and three-dimensional views. In addition, a pseudo-panoramic radiograph was created. For virtual implant positioning, appropriate implants were selected from a wide implant database from major international implant manufacturers and exchanged as often as required. In addition, an abutment was freely defined and virtually assigned to the implant. Abutment diameter, height, inclination, and rotation could be changed as required. The virtual abutment allowed the ideal implant position to be determined depending on the requirements that the implant axes must meet for an appropriate prosthetic restoration [8]. 

#### Transfer of virtual planning to the surgical site

Following virtual implant planning and referencing, data were transferred to a dental laboratory. Based on virtual planning, surgical guides were created using a gonyX table (IVS Solutions AG, Chemnitz, Germany) in the laboratory. 

After the templates had been produced and the exact drilling protocol had been printed, the preparation of the implant site and the implant insertion were performed in a template-guided manner. Following the administration of local anesthetics a flap was raised using a crestal incision and the drill guide was positioned appropriately. The implant bed was prepared in accordance with the manufacturer’s instructions. Titanium implants from Institut Straumann^®^ AG (Basel, Switzerland), Camlog^®^ Biotechnologies AG (Basel, Switzerland), and Dentsply^®^ IH (Mannheim, Germany) were used. All implants had a moderately rough, sandblasted, and acid-etched surface. 

The following techniques of simultaneous augmentation at the time of implantation were used:

- External sinus lift with simultaneous implantation (when residual vertical maxillary bone ≥5 mm)
- Internal sinus lift (by insufficient vertical maxillary bone when the apical implant part did not prospectively extend >2 mm into the maxillary sinus)
- Bone splitting
- Implantation and simultaneous applying of bone chips gained with a scraper device

#### Prosthetic restoration

Implant exposure was performed 3–4 months after implant placement. Prosthetic restoration and implant loading were initiated 2 weeks after implant exposure at the earliest. 

### Data collection

Data were collected from patients’ hospital charts and patients were anonymised before data analysis. Extracted data comprised: 

**Pre-surgical parameters**

- Patient’s age
- Medical history
- Smoking habits
- History of periodontitis
- Oral hygiene status (API and SBI) at the initial examination

The modified API and the modified SBI were collected in advance by the referring armed forces dentist to record oral health and plaque infestation objectively. In the API, after visualizing the plaque, a plaque elevator was used in a yes/no decision to assess whether plaque was present in the proximal space. The assessment was made in the first and third quadrants from palatal/lingual and in the second and fourth quadrants from buccal. When determining the SBI, the sulcus area is probed mesially and distally without pressure, in the first and third quadrants from buccal and in the second and fourth quadrants from palatal and lingual, respectively. In a yes/no decision, the presence of bleeding is assessed. Both indexes are given as a percentage. 

**Parameters of the augmentation and implantation phase**

- Augmentation procedures
- Recipient jaw area (maxilla/mandible, anterior/premolar region/posterior)
- Dental situation of the implant region (tooth gap, free-end dental arch, edentulous)
- Implant features (system, diameter, length, type) and implant position (maxilla/mandible and anterior/premolar region/molar region)
- Complications after augmentation
- Complications after implantation
- Bone graft survival prior to implant placement

Complications related to implant procedures were defined as follows:

**Intraoperative complications**

- Change of planning intraoperatively and free-hand implant insertion as a result of unfavorable implant position, reduced mouth opening, or fracture of the surgical template.

**Early postoperative complications**

- Wound healing complications of the recipient site: soft tissue dehiscence, bone graft exposure, wound infection
- Sensory disturbance at the neural supply area of alveolar inferior nerve, lingual nerve, and infraorbital nerve

**Late postoperative complications**

- Bone graft failure
- Early implant failure (before prosthetic restoration)
- Late implant failure (within 5 years after prosthetic restoration)
- Peri-implant mucositis
- Peri-implant bone resorption
- Peri-implantitis
- Technical complications related to prosthetic restoration: fracture of the framework, fracture of veneering
- Mechanical complications related to prosthetic restoration: abutment screw loosening, abutment screw fracture, crown loosening, crown loss, implant fracture

### Clinical and radiological 5-year follow-up

The patients were invited for a 5-year follow-up evaluation either by phone or in writing. The same clinician evaluated all participants. The referring dentist performed follow-up examinations during the 5-year period at unspecified intervals. Patients who could not attend the clinical examination in person were interviewed by phone where information on implant survival (implant in situ: yes/no) and technical complications was collected.

The following parameters were assessed:

- Peri-implant mucositis [21]:
  - Presence of bleeding and/or suppuration on gentle probing with or without increased probing depth compared to previous examinations
  - Absence of bone loss beyond crestal bone level changes resulting from initial bone remodeling
- Peri-implantitis:
  - Presence of bleeding and/or suppuration on gentle probing
  - Probing depths of ≥6 mm
  - Bone levels ≥3 mm apical of the most coronal portion of the intraosseous part of the implant
- Peri-implant bone resorption in mm
- Early and late implant failure

Digital radiographs were used to compare the peri-implant bone situation at the 5-year follow-up. For technical facilitation, only bone loss mesially and distally to the implant was measured. The ProVision PACS^®^ program (Cerner Corporation, Kansas City, USA) was used to assist measurement. A specialized technician did calibration every two years. The study coordinator carried out all radiographic measurements. To reduce measurement bias of the radiological right-angle projection technique, especially in the anterior maxilla, the following formula was used: 

Bone resorption = 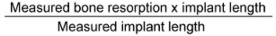


**Figure 3:**
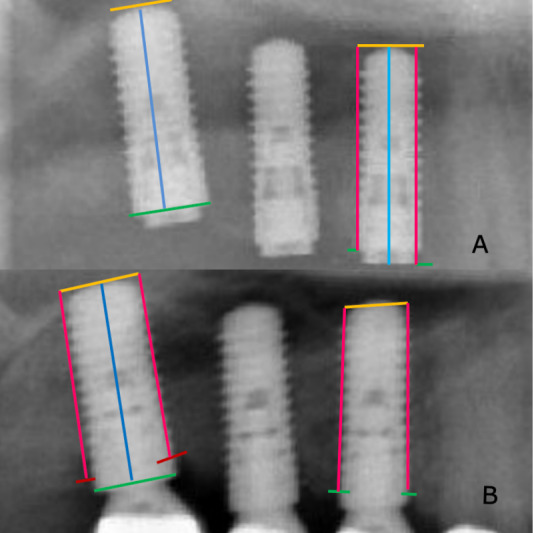
A: Postoperative radiograph after insertion of 3 implants in the posterior maxilla region with representation of the measurement procedure for determining the initial findings. B: Radiograph of the same area at the 5-year follow-up examination with representation of the measurement procedure for determining peri-implant bone resorption. Green line: initial bone height; blue arrow: implant length; orange line: apical implant edge; pink arrow: measured distance from the crestal bone to apical implant edge; red line: crestal bone line after peri-implant bone resorption

Bone resorption was defined as the distance between the implant shoulder and apical end as reference points. Implant length was defined as the distance from the radiologically visible implant shoulder to the apical end, parallel to the implant wall. Any discrepancy between the implant shoulder and the crestal bone line was considered when calculating bone loss. This formula was also used to assess vertical bone resorption on panoramics since the objects shown were increased by a factor of 1.4 (Figure 3 (Fig. 3)).

#### Implant success and survival

Implant success was based on the following criteria:

- No pain on function
- No implant mobility
- No history of exudation
- 0–2 mm peri-implant bone resorption radiologically

Cases with untreatable peri-implantitis were defined as implant failure. The implant survival rate was defined as the number of implants that were in situ at the 5-year follow-up (either clinically observed or via phone confirmed) divided by the number of inserted implants, the data of which could be evaluated at the follow-up examination.

#### Implant failure

Early and late implant loss were evaluated to define the clinical success of osseointegration. Early implant failure occurred between implant placement and abutment connection. Implants inserted after re-implantation were not included in the survival rate analysis. Late implant failures were documented within a period of up to 5 years after prosthesis loading and were defined as untreatable peri-implantitis with peri-implant bone resorption more than half of the implant length. Accordingly, the implant failure rate was calculated as the sum of early and late implant failure.

### Statistical analysis

Data were centralised in electronic format using Microsoft Excel software. Statistical analysis was performed using SPSS Statistics^®^, Version 23 (International Business Machines Corporation, Armonk, USA). Descriptive statistics were used to describe baseline patient characteristics. All categorical variables were expressed as absolute values (n) and relative prevalences (%). To determine significance, the parameters to be compared were presented in a cross-tabulation table. Chi-square tests were used to compare the frequencies of two nominal variables. The Fischer exact test was used in smaller subgroups, when the requirements for the chi-square were not met. Statistical significance was set at p<0.05. The potential impact of wound healing complications, augmentation procedures, smoking, history of periodontitis, and preoperative API and SBI values to peri-implant mucositis, peri-implant bone resorption, peri-implantitis, and early and late implant failure was investigated in a multivariable regression analysis. 

## Results

A total of 488 implants was inserted in 292 patients (275 men and 17 women) with a mean age of 33 years (IQR=19.25). Data were obtained for analysis from 229 (80.9%) patients with 368 (78.9%) inserted implants (Table 1 (Tab. 1)). Recipient jaw, tooth region, dental situation, implant dimensions, and the implant system used are presented in Table 2 (Tab. 2) and Table 3 (Tab. 3). The mean age of the patients who attended the follow-up was 38.5 years (IQR=17.0). 93% (n=236/253) of the implants were inserted in men and 6.7% (n=17/253) in women. One hundred and ninety-one (40.9%) of implant recipients were smokers. 

Augmentation before implantation was significantly more frequent in the maxilla (149/242; 61.5%) compared with the mandible (52/224; 23.2%) (chi-square test: p<0.001). The anterior maxilla was augmented in 94.8% and the anterior mandible in 100% of cases. The crista zygomaticoalveolaris and the mandibular symphysis were the main donor sites for grafting in the anterior maxilla and mandible. Augmentation of the premolar region was significantly more frequent in the maxilla than in the mandible (chi-square test: p=0.003). The posterior maxilla was augmented in 54.5% (n=54/99) of cases, mostly with grafts from the iliac crest, while the posterior mandible was augmented in 18.2% (n=30/164) of cases, mostly with bone grafts from the retromolar region. 

Within the cohort, 12 implants were placed without guide support despite computer-assisted three-dimensional planning due to intraoperative complications (Table 4 (Tab. 4)). 

### Surgical outcome

#### Early implant failure

Five implants in five patients had to be removed before restoration, resulting in an early implant failure rate of 1.07% (n=5/466). Four implants were removed because of insufficient osseointegration (biological complication) and one implant was removed despite sufficient osseointegration because the healing cap could not be removed after exposure (technical complication). Two implants were inserted in sides primarily grafted with bone from the crista zygomaticoalveolaris; one was inserted with simultaneous grafting of autologous bone chips. Two out of 5 implants were inserted in smokers, with history of periodontitis and API>20%. The patients with biological complications wanted no further implant treatment. The implant with technical complications was surgically removed and a new one re-inserted after an uneventful healing period. This case was not included in the statistical analysis. 

#### Late implant failure

All 368 implants were in situ in 229 patients who could be clinically and radiologically examined or interviewed via phone at the 5-year follow-up. Among the 271 clinically and radiologically examined implants, two implants with untreatable peri-implantitis and peri-implant bone resorption more than half of the implant length were defined as late implant failure (0.7%) (Table 5 (Tab. 5)). 

#### Implant survival

An overall implant survival rate of 98.1% (n=361/368) at the 5-year follow-up was revealed. 

78.2% (n=212/271) of implants showed no signs of peri-implant bone resorption. Twenty-eight (n=28/271; 10.3%) implants showed bone resorption of up to 1 mm and 23 (n=23/271; 8.5%) implants showed bone resorption of 1–3 mm. Radiological bone resorption ≥3 mm was detected in 1.8% of the implants examined. Among them, five implants showed bone resorption ≥3–4 mm and one implant showed bone resorption ≥4 mm (<1/2 implant length). Two implants presented untreatable peri-implantitis and peri-implant bone resorption more than half of the implant length (Table 5 (Tab. 5)). 

**Figure 4:**
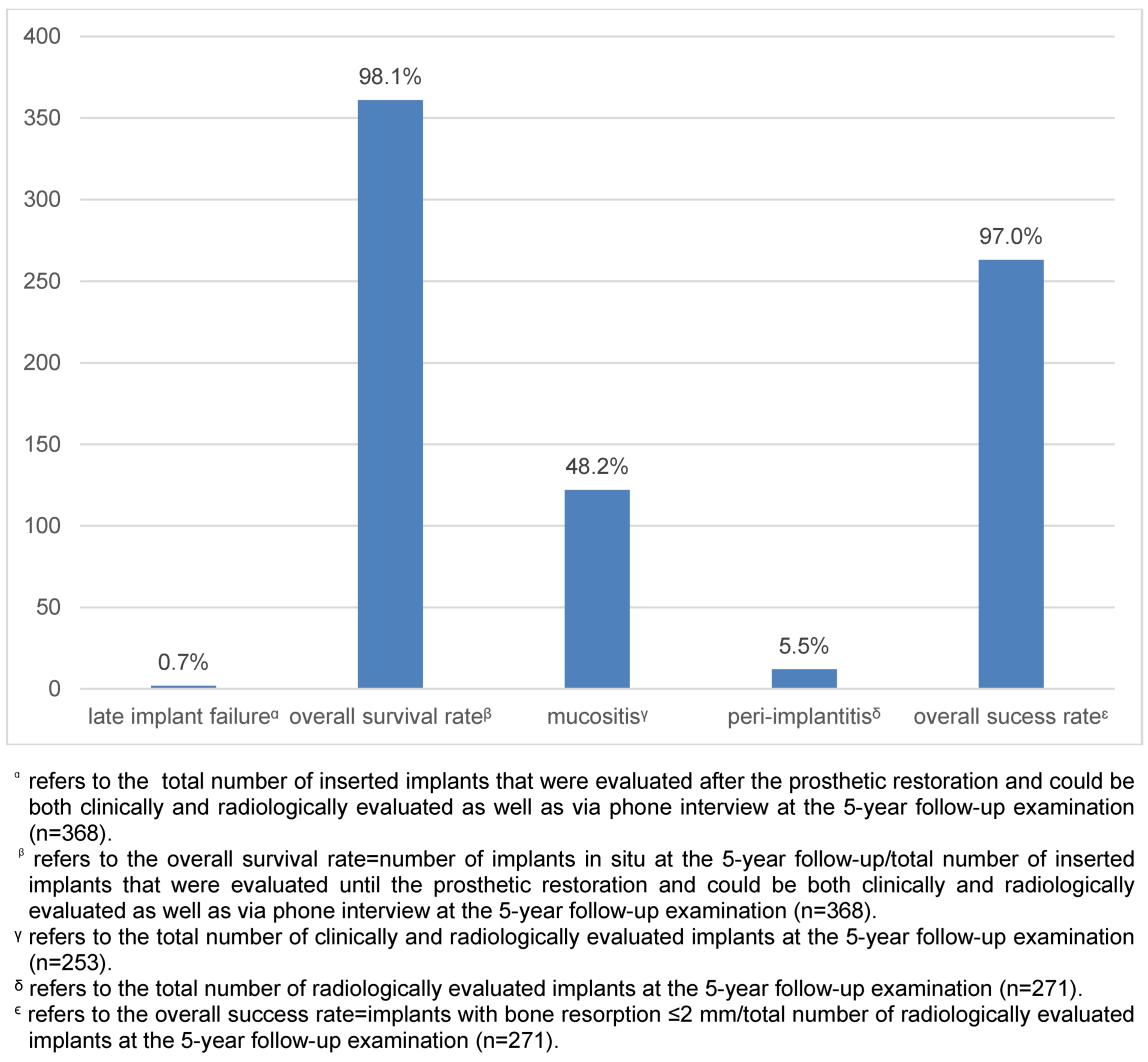
Outcome of prosthetically guided implant surgery at a 5-year follow-up examination

48.2% of implants were affected by peri-implant mucositis (n=122/253), detected at the clinical follow-up. Peri-implant bone resorption (median=0 mm, minimum=0 mm/maximum=7.3 mm) was detected in 21.7% of the radiologically examined implants (n=59/271). Fifteen cases of peri-implantitis (5.5%) were detected. The referring dentist already diagnosed seven cases during the 5-year period after loading, and eight were diagnosed at the follow-up examination (Figure 4 (Fig. 4)). In every case, peri-implantitis therapy was initiated in the same clinic. 

#### Implant success

According to the success criteria of the study, the implant success rate at the 5-year follow-up was 97.04% (n=263/271) (Figure 4 (Fig. 4)). 

#### Nerve damage

Persistent postoperative sensory disturbances were documented in three patients at the 5-year follow-up. In the first patient, in whom bone was harvested from the crista zygomaticoalveolaris, infraorbital nerve hypoesthesia was diagnosed. In the second patient, hypoesthesia of the right lingual nerve after bone harvesting from the mandibular ramus was documented. In the third patient, intermittent hypoesthesia of the left mental nerve was detected after augmentation of the posterior mandible with grafts from the iliac crest. There was no prevalence of nerve disturbances after bone harvesting from the mandibular symphysis or from the iliac crest, or after sinus lift procedures.

#### Wound healing complications after augmentation and implantation procedures

**Figure 5:**
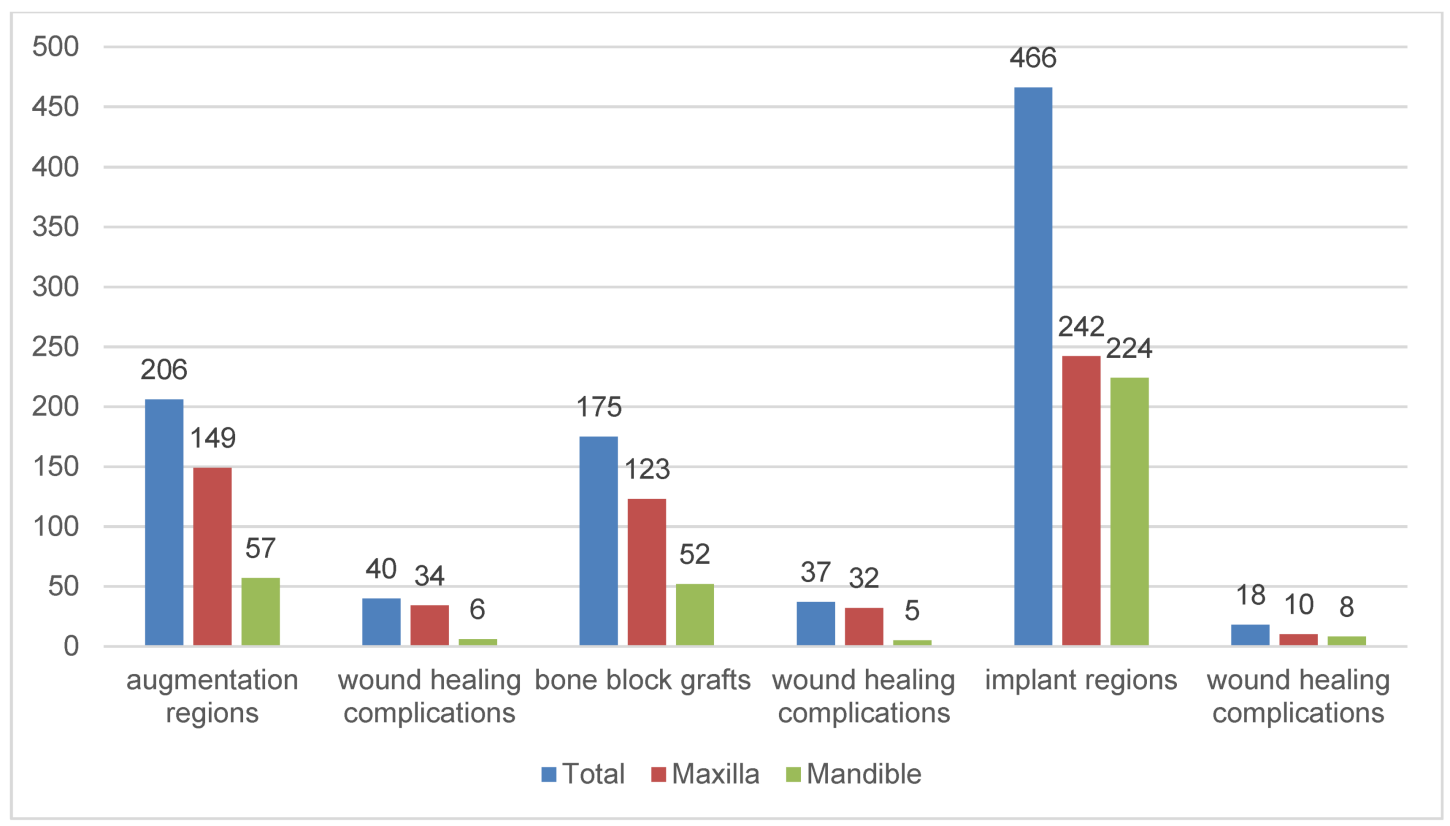
Distribution of wound healing complications after augmentation and implantation procedures in correlation to the recipient jaw

Two hundred and six implants (148 in the maxilla and 58 in the mandible) were inserted in 206 grafted sides. Wound healing complications occurred in 19.4% (n=40/206) of these implants. The maxilla (n=34/148; 22.9%) was significantly more frequently affected by complications than the mandible (n=6/58; 10.3%) when augmentation procedures were performed (chi-square test: p=0.049). Considering only the autologous bone grafts, complications were significantly more frequent in the maxilla (maxilla: n=32/123 and mandible n=5/52, chi-square test: p=0.015). In 466 implant regions, wound healing complications occurred in 18 (3.8%) regions, among them 10 in the maxilla and 8 in the mandible (chi-square test: p>0.999). The distribution of wound healing complications after augmentation and implantation procedures in correlation with the recipient jaw is presented in Figure 5 (Fig. 5). 

Hundred sixty-three out of 175 (n=163/175) block grafts were performed successfully, presenting a success rate of 93.1%. Twelve grafts (n=12; 6.9%) were surgically removed due to severe wound healing complications. Seven out of 60 iliac crest grafts, three of 37 mandibular ramus grafts, and two of 72 crista zygomaticoalveolaris grafts were lost. All six symphysis grafts were successful. Six iliac crest grafts were lost in the maxilla and were performed simultaneously to external sinus lift with cancellous bone from the same donor side. In four of 12 cases of graft failure, re-augmentation was performed. In the other cases, computer-aided planning showed sufficient bone volume despite graft removal and implants were inserted without further augmentation. Most wound healing complications occurred in crista zygomaticoalveolaris grafts (n=18/72; 25.0%) followed by iliac crest grafts (n=13/60; 21.6%). Complication rates of these two donor sites differed significantly from the others (chi-square test: p=0.012 and p=0.019, respectively). 

Wound healing complications after implantation were diagnosed in 3.9% (n=18/466) of the cases during the healing phase. Augmentation was performed in 12 of these cases, in 10 cases simultaneously to the implant insertion and in two cases pre-implantologically. External sinus lifts with simultaneous implantation (n=2/10; 20%) and implantations with local grafting of bone scrapes (n=7/43; 16.3%) significantly increased wound healing complications compared to internal sinus lift (n=1/45; 2.2%) and bone splitting (n=0; 0%) (chi-square test: p<0.001). Wound healing complications after implantation in the maxilla (n=10/18; 55.5%) and the mandible (n=8/18; 44.5%) did not differ significantly from each other (chi-square test: p>0.999).

#### Technical and mechanical complications

Forty-one technical and seven mechanical complications were documented in the follow-up examination within the 5-year period after prosthesis loading. No implant fracture occurred (Table 6 (Tab. 6)). 

### Multivariable analysis

The impact of variable factors on the development of peri-implant mucositis, peri-implant bone resorption, peri-implantitis, and early or late implant failure was statistically analyzed and demonstrated in Table 7 (Tab. 7).

**Figure 6:**
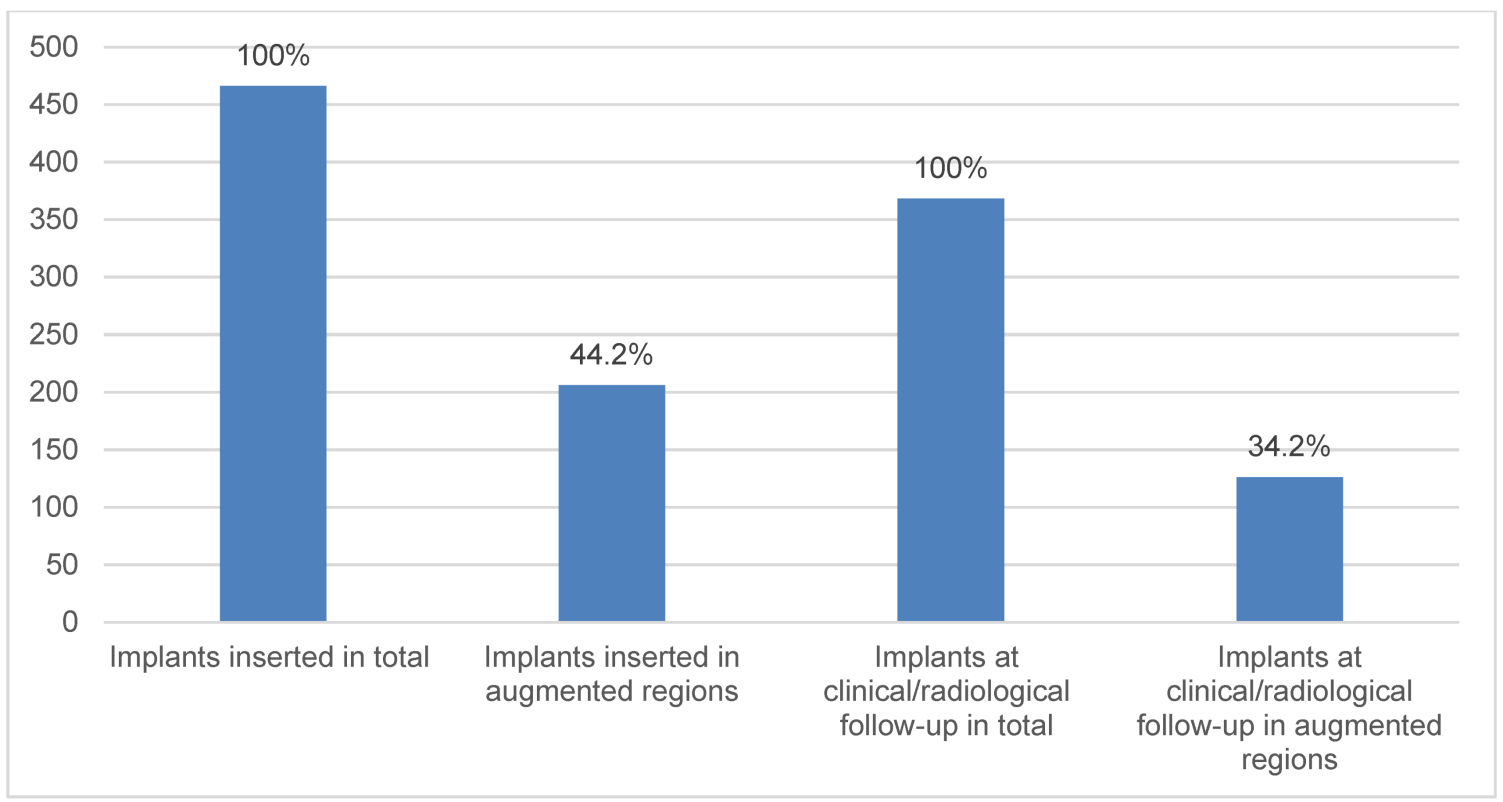
Demonstration of the implant number inserted in total and implant number evaluated at the 5-year follow-up with regard to pre-implantological augmentation procedures

At the 5-year follow-up, 61.2% (n=126/206) of augmented implant regions could be analyzed (Figure 6 (Fig. 6)). Peri-implant bone resorption was significantly higher after augmentation (p=0.028). However, augmentation procedures did not have a significant effect on peri-implant mucositis and peri-implantitis. 

To evaluate the impact of wound healing complications on implant outcomes, 28 out of 40 (70%) wound healing complications after augmentation procedures and 12 out of 18 (66.6%) wound healing complications after implantation were analyzed. No significant impact of wound healing complications after pre-implantological augmentation procedures with regard to the development of peri-implant mucositis, peri-implantitis, peri-implant bone resorption, and implant failure was demonstrated. However, wound healing complications after implantation significantly increased early implant failure (p<0.001). 

Regarding implants placed in smokers, both the prevalence of peri-implant bone resorption and peri-implantitis were statistically significant. In 84 implants placed in smokers, radiographically visible bone resorption was detected in 25 implants (29.8%) (non-smokers: 16.8% (28/167)). Peri-implantitis was diagnosed in 4 out of 83 implants placed in smokers, while one implant was affected in non-smokers (1/167). The prevalence of peri-implant mucositis was statistically insignificant when comparing implants placed in smokers and non-smokers.

Implants in patients with API>20% (n=42/72; 58.3%) presented significantly higher rates of peri-implant mucositis compared to those inserted in patients with API≤20% (n=72/159; 45.2%). However, there was no evidence of an increased risk of peri-implant bone resorption, peri-implantitis, and early or late implant failure with an API>20%. History of periodontitis and SBI>20% showed no increased risk within the parameters studied. 

## Discussion

Prosthetically guided oral implantology using computer-assisted 3D planning and template-guided implant surgery has been the focus of attention of researchers in the recent years due to its numerous advantages for dental implantation [17]. The present study assesses the 5-year surgical outcome of this concept in terms of implant survival and success rate and evaluates the impact of clinical variables on the development of peri-implant mucositis, peri-implant bone resorption, and peri-implantitis in a medium-term follow-up period. 

Prosthetically driven surgery is assumed to be accurate, precise, and reliable and enables an efficient patient-centric treatment workflow [4]. Three-dimensional reconstructions and multiplanar cross-sections, oriented along the alveolar process in the implant region, facilitates an ideal implant position and accordingly the position of the final crown due to diagnostic cast wax up [14]. The key point to enhance the precision of guided surgery is an accurate transfer of the position of 3D planned implants to the patient’s mouth [17], [22]. Individual abutments are also beneficial for aesthetics because the shape of the emergence profile can be individually designed and adjusted with respect to the prosthetic set-up [14], [23]. In case of an unfavorable abutment position, its visualization at the time of the prosthetic set-up and virtual implant planning helps to improve the implant position and selection of components [14], [23]. The selection of an implant planning software is therefore dependent on the specific implant system used in the daily routine [10]. 

The early implant failure rate in this study was 1.07%, which is low compared with similar studies [24], [25], [26], [27], [28]. Kang et al. documented an early survival rate of 95.5% evaluating 1,031 implants in 409 patients [24]. Chrcanovic et al. reported early implant failure in 1.74% of 10,096 implants and demonstrated smoking as significant influence factor [25]. In a large Swedish population, an early failure rate of 1.4% before prosthetic restoration was documented [27]. Brügger et al. also observed a low early loss of 0.6% in 1,568 implants, similar to a previous study from the same institution with a loss of 0.7% reported [26], [28]. In the present study, three out of four implant loss cases had been previously augmented. Among them, two patients had a history of periodontitis, two patients were smokers, and one patient had unfavorable API and SBI values preoperatively. In their meta-analysis Manzano et al. have already reported the impact of smoking, surgeon’s experience, implant region, poor oral hygiene, periodontitis history, and wound healing complications on early implant failure [29]. According to the authors’ experience, several factors could contribute to early implant failure, however, a significant correlation of pre-implantological augmentation procedures, wound healing complications, smoking, history of periodontitis, and preoperative API and SBI≥20% with early implant failure was not detected in this study. However, the low case number of early implant failure was not sufficient for a valid statistical analysis. Nevertheless, these clinical factors have to be considered pre-implantologically to prevent complications.

In this study, implants with untreatable peri-implantitis and bone resorption greater than half the implant length were defined as late implant failure. Accordingly, a success rate of 97.04% and an overall implant survival rate of 98.1% was documented. These results are in concordance with the current literature [30], [31], [32]. Yogui et al. reviewed four studies that included 154 patients with 597 dental implants and reported an implant survival rate of over 95% in a mean follow-up period of 2.25 years [30]. Derksen et al. reported a 99.3% survival rate 2 years after guided insertion of 145 implants in 66 patients [31]. Urban et al. reported an implant survival rate of 100% 76.5 months after 122 dental implants were inserted into augmented sites, demonstrating the reliability of this treatment concept [32]. Prospective long-term studies and a more standardized recall protocol should validate the promising but medium-term results of the present study. 

Mucositis in peri-implant soft tissues has already been investigated in various follow-up intervals with reported prevalences ranging from 21% to 56% [33], [34], [35], [36], [37]. The current 5-year follow-up examination showed mucositis in 48.2% of the examined implants, which is within the reported range. 

1–2 mm bone resorption in 8.5% and ≥3 mm bone resorption in 2.9% of the examined implants was observed, which is favorable compared with recent studies [32], [38], [39]. Doornewaard et al. analyzed 225 studies and documented an average bone resorption of 1.1 mm in 23% of 8,182 implants in a 5-year follow-up [38]. Urban et al. reported a mean peri-implant bone loss of 1.4±1.0 mm, which is consistent with implant success [32]. Wennström et al. also showed similar values for bone resorption as in the present study (11.1%) [39]. In the current study, peri-implantitis was diagnosed in 5.5% of the implants, which is lower than previous reported rates (8.9–12%) [34], [35], [36], [37], [40]. French et al. diagnosed peri-implantitis in 4.7% of cases 6–7 years after loading, while Jepsen et al. demonstrated a prevalence of 22% in their meta-analysis [34], [35]. A separate analysis of the correlation of clinical findings, such as probing depth, bleeding on probing, and PSI value with the development of mucositis, bone resorption, and peri-implantitis, was not aim of this study. The effectiveness of the peri-implantitis therapy was also not evaluated. 

Wound healing complications after augmentation did not affect peri-implant mucositis, peri-implant bone resorption, peri-implantitis, and implant failure significantly in this study. These complications were significantly more frequent in the maxilla. The low prevalence (3.9%) of wound healing complications after implantation increased significantly only regarding the late implant failure rate among the studied parameters. Considering that the prevalence of wound healing complications after preoperative augmentation was significantly affected by smoking, history of periodontitis, and API>20%, patients with these features should be informed in advance about the increased risk of treatment failure. 

One of the postulated advantages of prosthetically guided surgery is the occasional avoidance of augmentation procedures due to preoperative virtual planning [6]. Several works have been published favorizing guided surgical protocols in atrophic areas without pre-implantological augmentation [6]. Fortin et al. evaluated a protocol of CAD/CAM surgical template based on digital planning, exploiting anterior or posterior wall or the septa of the sinus as well as the palatal curvature. They reported 98% implant survival rate after 4 years in partially edentulous cases with severely resorbed posterior maxilla avoiding sinus augmentation procedure [16]. In the present study, augmentation procedures were performed when after virtual planning a primary implantation in every possible angulation was not feasible. 

The study demonstrated a significantly increased peri-implant bone resorption after augmentation, however, an impact on peri-implant mucositis, peri-implantitis, and implant failure was not detected. Implants in grafted regions of the mandible showed higher bone resorption at the 5-year follow-up than in the maxilla. Among the autologous grafts harvested, retromolar grafts for mandibular reconstruction led to significantly higher peri-implant bone resorption. Tran et al. investigated the influence of bone grafts on implant survival in a 5- and 10-year follow-up examination and found no significant differences between the different grafts [41]. Sbordone et al. also found no differences in implant survival between augmented and native bone, however, peri-implant bone loss differed between implants inserted in augmented and native bone [42]. His results are in concordance with the present study. The use of autologous bone in this study resulted in excellent graft survival and success rates with low complication rate, representing a viable treatment option with predictable grafting and implantation success. This is supported by recent studies [15], [43], [44], [45]. The present study reported a higher implant survival rate in autologous grafted areas within 5 years after prosthesis loading compared with previous researches [46], [47], [48]. Additionally, the authors recommend a maximum healing period of 3–4 months after autologous bone grafting. Despite these results, a lack in the literature exists regarding to the impact of simultaneous bone chips augmentation on the implant survival and success rate. Rammelsberg et al. described a slight reduction in the impact of complications on implant survival when augmentation techniques were used simultaneously with implantation [49]. The evaluation of simultaneous augmentation procedures and soft tissue management after augmentation was not the aim of this study and should be evaluated in future studies with long-term follow-up protocols after prosthesis loading. 

It is postulated that guided surgery is correlated with reduced surgical complications [50]. In this study, intraoperative surgical complications included inaccurate virtual planning of the implant position, reduced mouth opening, and fracture of the surgical guide. In all cases, implants were successfully inserted in free-hand manner. Technical and mechanical complications of the prosthetical restoration at the follow-up examination were also reported, however, without influence to the implant outcome. In contrast to Cassetta et al., other researchers highlight the important number of complications, especially after prosthesis, of guided surgery protocols [51]. In their review Tahmaseb et al. reported intraoperative or prosthetic complications in 36.4% of the treated cases, including surgical complications like guide fractures or prosthetic complications like misfits with frameworks and prosthetic fractures [52]. The authors agree with Al Yafi et al. that the reliability of computer-guided surgery does not justify a blind execution. The learning curve is undeniable and a clinician with skills in conventional implant dentistry will easily be able to manage unexpected complications [4]. Similarly, Cassetta et al. consider furthering clinicians’ experience in conventional implant placement as essential before switching to guided surgery [53]. Colombo et al. in their review based on only two randomized controlled trials (RCTs) concluded that implant survival rate and effectiveness are similar for conventional and digital implant placement procedures after a follow-up period of at least 6 months [6]. The benefits that prosthetically guided surgery could provide compared to conventional protocols with digital workflows should be investigated in future RCTs. Reduction of postoperative pain and decreased surgical time have to be discussed, but the initial financial investment, the digital work flow costs for each clinical case, and the time for virtual planning should also be considered [6]. If guided surgery can avoid bone grafting procedures and reduce the treatment time significantly, it can reduce the overall treatment cost too [6]. Agreeing with Colombo et al., the authors recommend good preparation of the clinicians with regard to both new digital and conventional procedures und suggest further scientific research focused on the identification of clinical situations that could benefit most from guided surgery. 

There are some limitations to the current study. The retrospective nature of the research could lead to documentation bias and thus limit the generalizability of the results. Through this retrospective review, only prevalences and correlations could be described. However, the authors believe that this limitation is clearly outweighed by the large implant collective. Furthermore, the majority of patients was healthy and in working age, so age-specific delayed wound healing or medication-induced influence on the oral flora were absent, providing favorable conditions for implant treatment. An important limitation of this study is the quite old patient data set, collected from the years 2008 to 2010. The 5-year follow-up examination took place in years 2015 to 2016 and represents a medium-term follow-up. A longer long-term follow-up of at least 10 years to date could provide more reliable and representative information about the surgical outcome of the prosthetically guided implant concept. However, such an extended follow-up examination was not possible in the military hospital because of the professional status of the patients. A longer than 5-year follow-up period could increase the drop-out ratio and lead to bias, since some patients could have been transferred to other units, others might not attend the appointments due to military missions, and others could have been discharged from their military service. Although only 58.2% of the inserted implants were included in the 5-year follow-up, the study results obtained are representative of the total number of implants inserted, since the investigated parameters did not differ in the two groups. Only the proportion of implants placed in smokers was underrepresented at follow-up. Since smoking increases the risk of complications, it is possible that further cases of peri-implantitis occurred. Errors in retrospective data acquisition and clinical radiological follow-up were minimized because only one clinician obtained and documented these data. Consequently, there were no discrepancies in the evaluation of clinical and radiological parameters and their interpretation. The absence of a control group with patients undergoing bone augmentation procedures with bone substitutes also limits interpretation of the findings. The undocumented soft tissue management after implantation with possible influence on the development of mucositis, peri-implant bone loss, and periimplantitis could also limit the interpretation of the study results. 

## Conclusion

This study showed a high implant survival and success rate in a 5-year follow-up, confirming the reliability of prosthetically guided implant surgery, which combines computer-assisted 3D planning and template-guided insertion together with augmentation procedures when necessary. Intraoperative complications and technical or mechanical complications after prosthesis loading remain within acceptable clinical limits. The rate of peri-implant mucositis, peri-implant bone resorption and peri-implantitis was within the current literature range. Smoking increased the development of peri-implant bone resorption and peri-implantitis significantly, while post-implantological wound healing complications were correlated with higher early implant failure rate. Smokers and patients with inadequate oral hygiene and history of periodontitis should be informed in advance about the high complication risk to optimize the treatment outcome. Further studies should be designed as RCTs to clarify the clinical indications and investigate the long-term surgical outcome of prosthetically guided surgery.

## Abbreviations

- CT: computer tomography
- CBCT: cone-beam computer tomography
- API: approximal plaque index
- SBI: sulcus bleeding index
- IQR: interquartile range

## Notes

### Availability of data and materials

The datasets used and analyzed during the present study are available from the corresponding author on reasonable request.

### Ethics approval and consent to participate

Patient recruitment and data collection for this study took place at the Department of Maxillofacial and Facial Plastic Surgery at the military/academic hospital of Ulm University, Germany. Ethical approval for this study was obtained from the ethics committee of the chamber of physicians at Ulm University (Number: 85/14-Fa/se.). The research was conducted in full accordance with ethical principles, including the World Medical Association Declaration of Helsinki and its later amendments. The patients’ data were anonymized and de-identified prior to analysis. For this type of study, formal consent was not required. Reporting was based on the recommendations of the “Strengthening the Reporting of Observational Studies in Epidemiology (STROBE)” initiative [54].

### First authorship

Andreas Sakkas and Stefan Westendorf share the first authorship. 

### Authors’ contributions

Stefan Westendorf planned the design, coordinated the study, and carried out the data selection. Andreas Sakkas and Stefan Westendorf drafted the manuscript. Sebastian Pietzka and Oliver Thiele helped in editing the manuscript. Frank Wilde participated in the study coordination and helped in drafting the manuscript. Alexander Schramm contributed to the protocol preparation and guidance of the study and was involved in drafting the manuscript and finalizing it for submission. All authors besides Oliver Thiele operated on a part of the study patients. All authors read and approved the final manuscript.

### Competing interests

The authors declare that they have no competing interests in regard to this article and no financial interests, either directly or indirectly, regarding the products listed in the study.

### Acknowledgments

We would like to thank Bacon Editing^®^ for language editing this manuscript. We also thank the patients for their kindness to participate as study cases and the whole medical team at the military hospital of Ulm in Germany.

## Figures and Tables

**Table 1:**
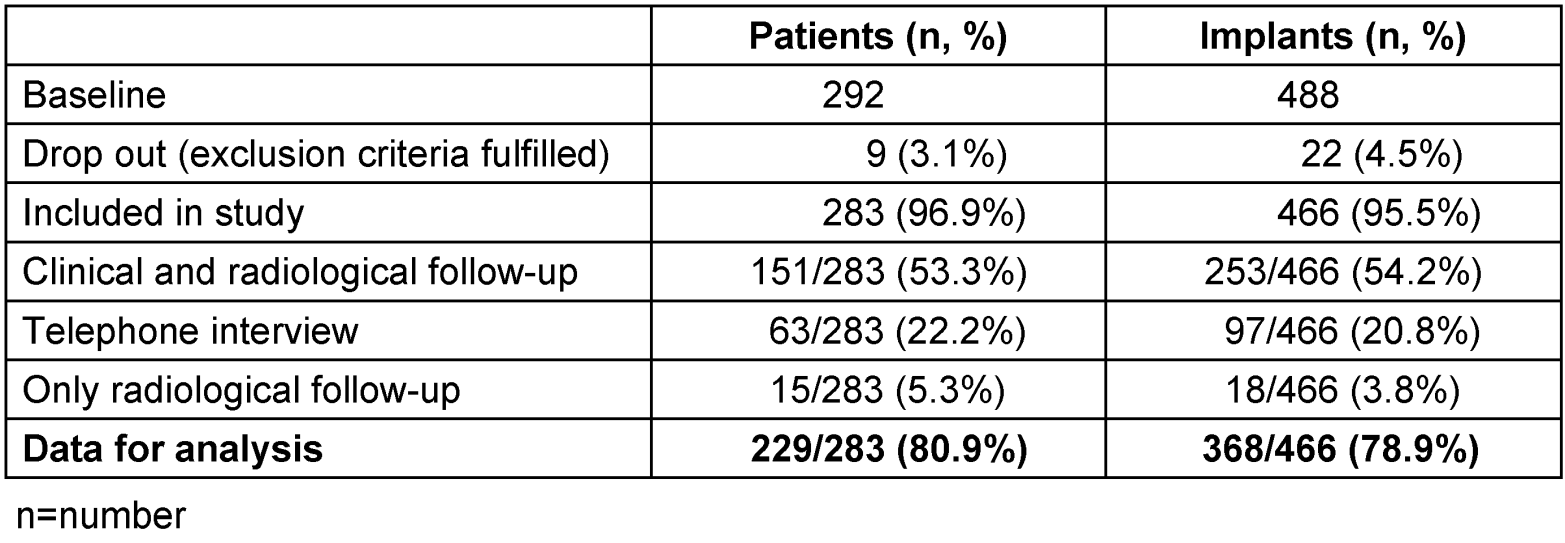
Study collective

**Table 2:**
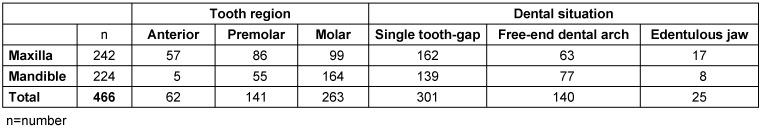
Implant number regarding the recipient jaw, tooth region, and dental situation

**Table 3:**
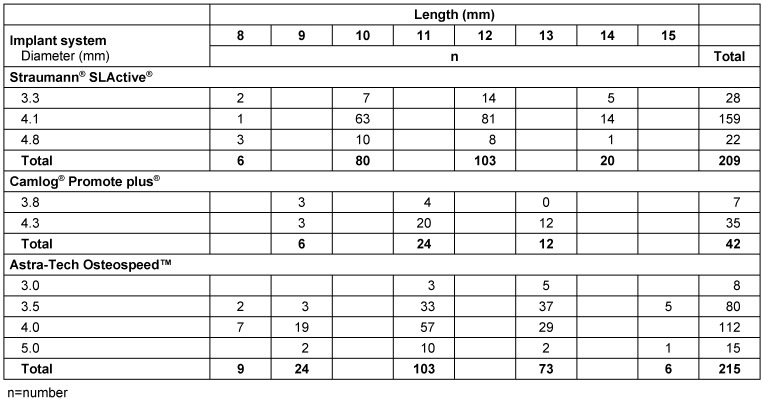
Distribution of implant dimensions and implant system used

**Table 4:**
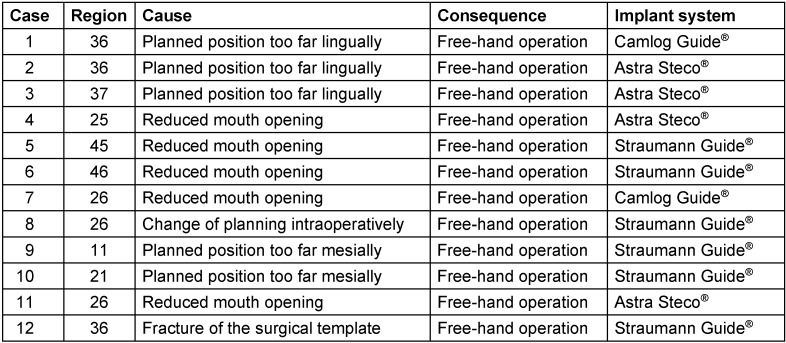
Cases of free-hand implantation due to intraoperative complications despite preoperative computer-assisted three-dimensional planning and surgical template support.

**Table 5:**
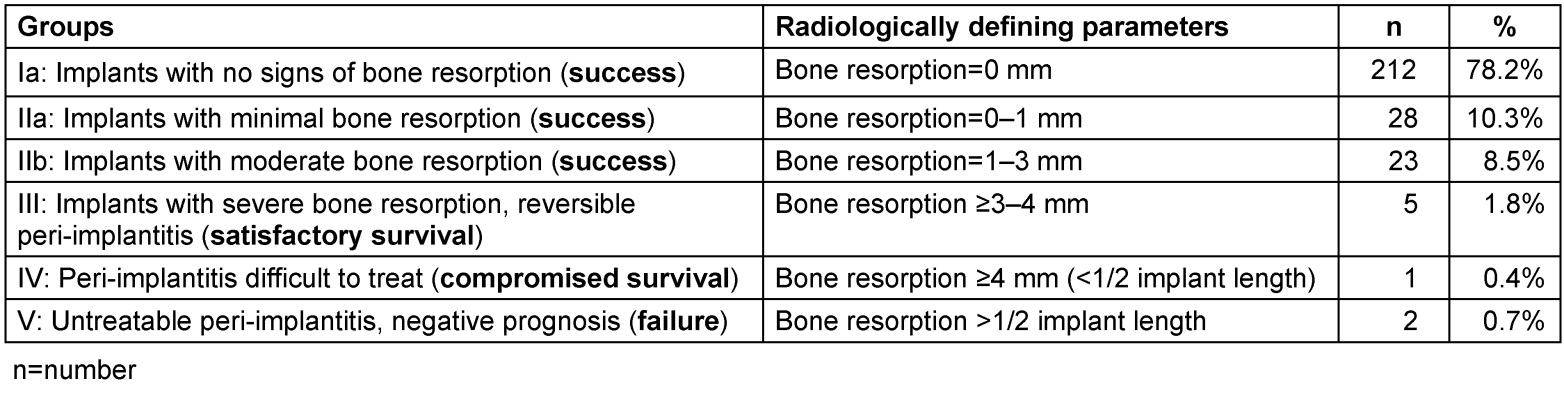
Success rate of the 271 implants examined at the 5-year follow-up with regard to peri-implant bone loss according to our success criteria, based on the criteria defined in the 2017 World Workshop on the Classification of Periodontal and Peri-Implant Diseases and Conditions [21]. The treatment success in our study was defined for a peri-implant bone resorption ≤2 mm.

**Table 6:**
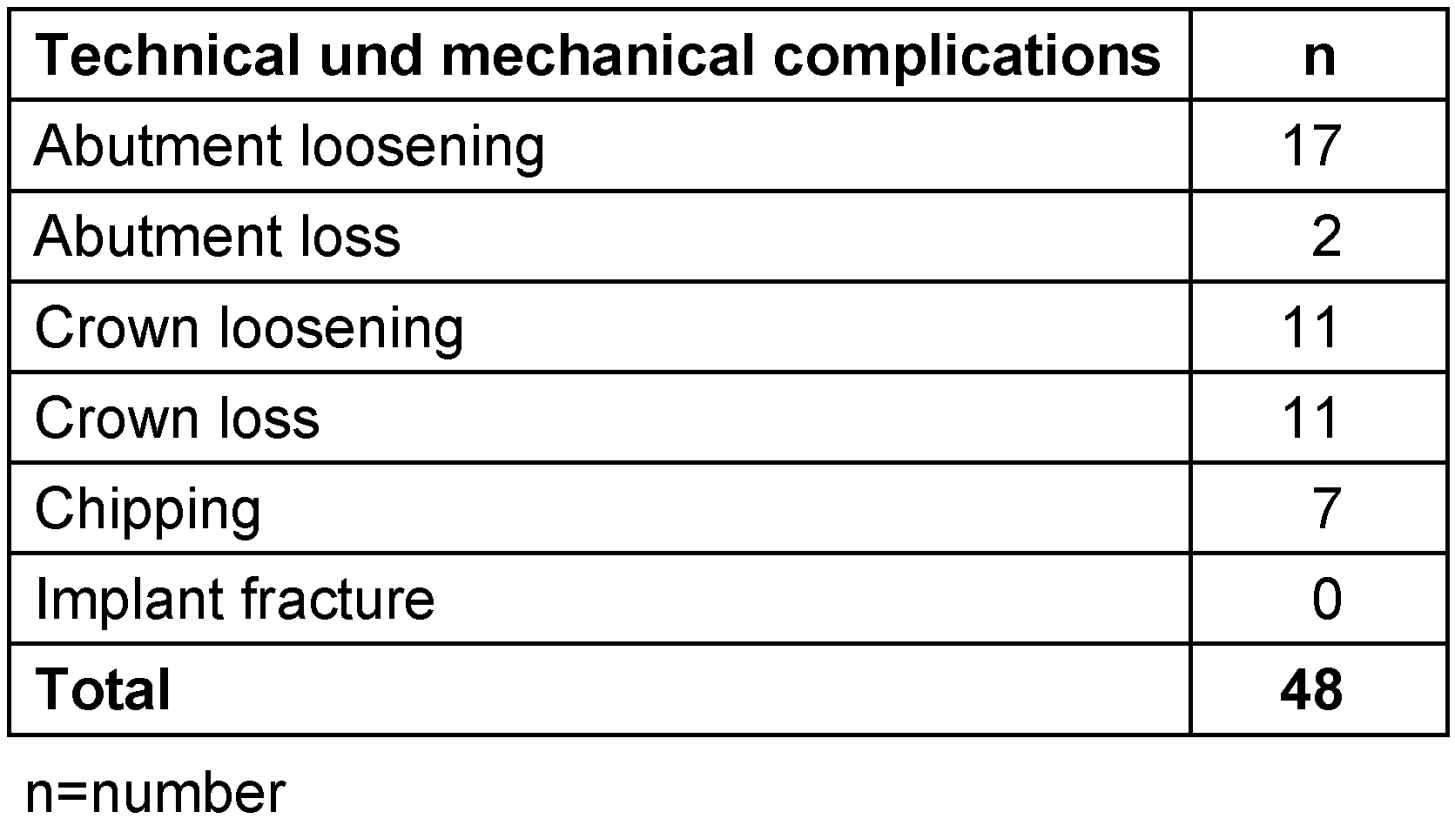
Technical and mechanical complications in the 5-year period after prosthesis loading

**Table 7:**
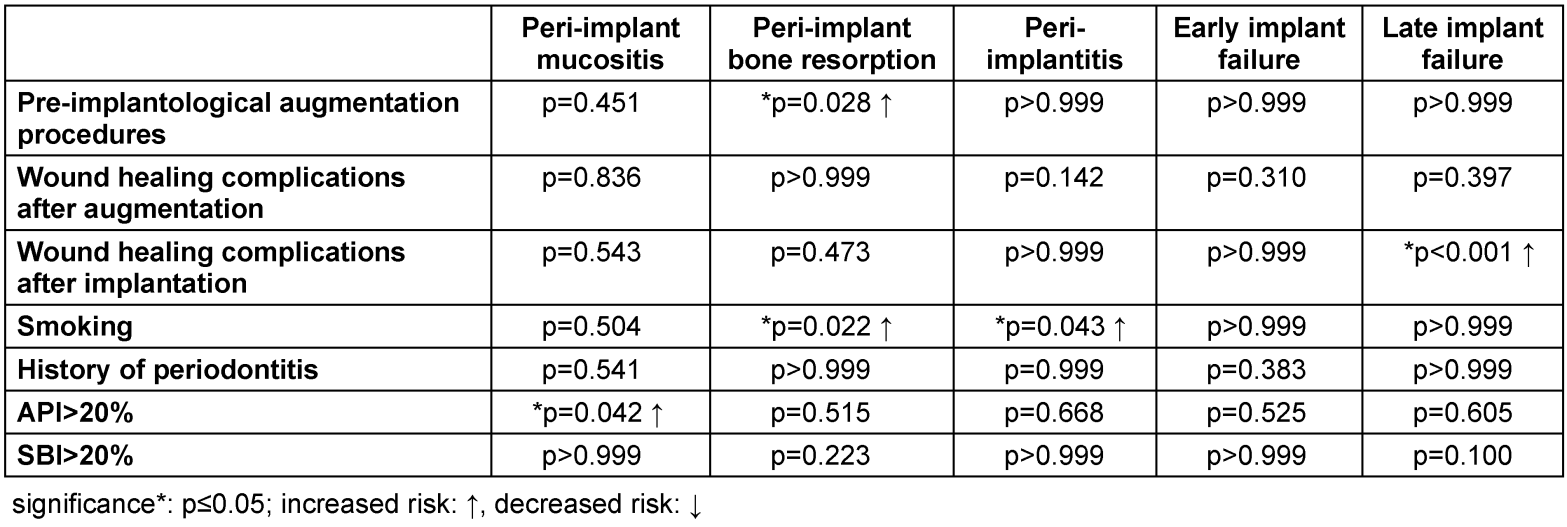
Statistical correlation between variable factors and the prevalence of peri-implant mucositis, peri-implant bone resorption, peri-implantitis, and implant failure
